# Spatial and Temporal Variability of Plant Leaf Responses Cascade after PSII Inhibition: Raman, Chlorophyll Fluorescence and Infrared Thermal Imaging

**DOI:** 10.3390/s20041015

**Published:** 2020-02-13

**Authors:** Petr Vítek, Barbora Veselá, Karel Klem

**Affiliations:** Global Change Research Institute of the Czech Academy of Sciences, Bělidla 986/4a, 603 00 Brno, Czech Republic; vesela.b@czechglobe.cz (B.V.); klem.k@czechglobe.cz (K.K.)

**Keywords:** Raman mapping, carotenoids, zeaxanthin, photosynthesis, oxidative stress, xanthophyll cycle, photoinhibition

## Abstract

The use of photosystem II (PSII) inhibitors allows simulating cascade of defense and damage responses, including the oxidative stress. In our study, PSII inhibiting herbicide metribuzin was applied to the leaf of the model plant species *Chenopodium album*. The temporally and spatially resolved cascade of defense responses was studied noninvasively at the leaf level by combining three imaging approaches: Raman spectroscopy as a principal method, corroborated by chlorophyll *a* fluorescence (ChlF) and infrared thermal imaging. ChlF imaging show time-dependent transport in acropetal direction through veins and increase of area affected by metribuzin and demonstrated the ability to distinguish between fast processes at the level of electron transport (1 − *V_j_*) from slow processes at the level of non-photochemical energy dissipation (NPQ) or maximum efficiency of PSII photochemistry (*F*v/*F*m). The high-resolution resonance Raman images show zones of local increase of carotenoid signal 72 h after the herbicide application, surrounding the damaged tissue, which points to the activation of defense mechanisms. The shift in the carotenoid band indicates structural changes in carotenoids. Finally, the increase of leaf temperature in the region surrounding the spot of herbicide application and expanding in the direction to the leaf tip proves the metribuzin effect on slow stomata closure.

## 1. Introduction

Most environmental stresses, such as drought, heat, salt, UV radiation, heavy metals or cold enhance the extent of photoinhibition, which is determined by the balance between photodamage to photosystem II (PSII) and its repair [1]. Environmental stresses mainly affect the repair of PSII through an inhibited synthesis of PSII proteins, particularly D1 protein. Inhibition of light-driven processes under stress conditions, comprising both energy transfer and electron transport results in formation of reactive oxygen species (ROS), which act as signaling molecule triggering a series of defense mechanisms or cause cellular damages, which are manifested in the form of degradation pigments, proteins, lipids, carbohydrates and DNA, and which ultimately amalgamate in plant cellular death [2]. A very similar cascade of processes can be, however, triggered also artificially by application of herbicides inhibiting both PSI and electron transport and resulting in ROS production [3]. It allows us to simulate such processes in the exact spatial and temporal manner and also to test the non-invasive imaging methods with the potential to evaluate the dynamics and spatial variability of defense and damage responses to environmental stresses.

A pigment-protein complex PSII that converts light to chemical energy is particularly sensitive to photoinhibition [4,5] and subsequent oxidative stress, which inhibits the repair of PSII [6]. One of the most promising tools for monitoring the photoinhibition represents chlorophyll fluorescence and particularly the maximum quantum yield of PSII (*F*v/*F*m; [7]), which allows also to monitor the spatial distribution of photoinhibition within the leaf and plant or to monitor multiple plants at the same time when chlorophyll fluorescence imaging is applied [8].

The damage of PSII proteins is closely linked with the occurrence of carotenoids and their capacity to scavenge ROS [9]. Carotenoids are pigments involved in photosynthesis. They have a photoprotective function and act also in membrane stabilization. Their chemical structure is characterized by a polyenic chain (conjugated double-bonds) composed of isoprenoid units. A known key function of xanthophyll carotenoids is dissipation of excess excitation energy in the xanthophyll cycle, which is the common photoprotective mechanism in plants and algae [10,11]. Zeaxanthin converted from violaxanthin during xanthophyll cycle enhances thermal energy dissipation, which is registered as non-photochemical quenching of fluorescence (NPQ) and direct scavenging of reactive oxygen species if any is formed [12,13]. Similarly to other chlorophyll fluorescence parameters, NPQ can be monitored with high spatial and temporal resolution using chlorophyll fluorescence imaging technique [14].

Raman spectroscopy is particularly sensitive to carotenoids due to the conjugated polyene structure [15], primarily when the green excitation wavelength is employed. It coincides with the absorption band of an allowed *π*–*π*∗ electronic transition, resulting in the resonance Raman effect [16,17,18,19]. It has been applied in plant science (e.g., [20,21,22,23]), including monitoring of the response towards early abiotic stress factors [24]. The typical Raman features of carotenoids are related mainly to the polyene chain [16,17]. The vibrational stretching mode of ν_1_(C=C) and ν_2_(C–C) is located in the region 1490–1540 cm^−1^ and 1150-1160 cm^−1^, respectively. The band of medium intensity corresponding to the rocking vibrations of methyl groups (δ(C-CH_3_)) occur at 1000–1010 cm^−1^ [16]. Raman imaging is an application of Raman spectroscopy that allows constructing chemical maps based on the Raman signal in each pixel of the map. It has become an important non-invasive tool in diverse analytic fields including applications in life sciences. Carotenoids within plant leaves were studied using the NIR-FT Raman technique by Baranski et al. [25] or Schulz et al. [26]. Dispersive Raman systems using a laser excitation in the visible range allow for imaging with high spatial resolution as applied, for example, to image carotenoid crystals in carrot cells [27]. Benefiting from the resonance Raman effect, this technique was applied by Vitek et al. [28] for imaging of carotenoid decline in sunflower leaves caused by herbicides based on the carotenoid biosynthesis inhibition mode-of-action.

The critical plant hormone mediating plant responses to both abiotic and biotic stress is abscisic acid (ABA), which acts as a signaling molecule in plant acclimation to stress or regulation of physiological responses such as stomatal closure or induction of plant senescence [29]. ABA biosynthesis is closely associated with carotenoid biosynthetic pathways. It starts in the plastids from the carotenoid zeaxanthin and ends in the cytosol with the formation of abscisic aldehyde, which is oxidized into ABA [30]. One of the first physiological responses following the increased ABA level is a closure of stomata and reduced transpiration rate together with a lower CO_2_ assimilation rate. Dynamics and spatial changes in transpiration rate can be monitored using infrared thermal imaging technique, which in recent years found a considerable expansion in remote sensing [31], ecophysiology [32], plant phenotyping [33] or stress physiology [34]. Although the resulting leaf temperature is an integration of several environmental factors and processes taking place inside the leaf, under well-defined conditions it can serve as a unique imaging tool for monitoring the stomatal responses [35].

Metribuzin represents one of currently most widely used PSII inhibiting herbicides. It has flexible use for pre- and post-emergence control of grasses and broad-leaved weeds in a variety of crops including cereals, legumes or potatoes. A better understanding of its behavior in a plant and the environment is therefore essential to improve efficacy, reduce crop damage, reduce impacts on non-target organisms and to minimize the persistence in the environment, particularly in the soil and surface- or ground-water. Metribuzin also exhibits an inhibitory effect at very low concentrations when applied on sensitive weed species such as *Chenopodium album*, L. [36]. The optimization of metribuzin use from the perspective of herbicide dose, application conditions, weed growth stage, spraying technique or use of adjuvants may thus benefit from using chlorophyll fluorescence, Raman and thermal infrared imaging techniques.

In this study, we employed these non-invasive imaging techniques to monitor various biochemical and physiological responses, which are common to both PSII inhibitors effect and a range of abiotic and biotic stresses, to detect the cascade of responses, their dynamics and also spatial distribution within the plant leaves. These techniques may find application in high throughput screening of genotypes resistant to abiotic or biotic stress—plant phenotyping, in understanding and stimulating the processes leading to induction of defense mechanisms, or in optimizing the herbicide application.

## 2. Materials and Methods

### 2.1. Plant Material and Herbicide Treatment

*Chenopodium album* L. seedlings were grown in FS-4600 growth chambers (Photon Systems Instruments spol. s r.o., Drásov, Czech Republic) under a 15 h day/9 h night regime. The temperature, relative air humidity and intensity of photosynthetically active radiation were changed gradually with day/night maxima and minima 60%/80%, 25/15 °C and 1000/0 μmol m^−2^ s^−1^ respectively. The light was provided by super bright white LEDs. Plants were grown in pots of size 11 cm × 11 cm × 12 cm filled with horticultural substrate (mixture of peat and humus) providing necessary nutrients for the first month of growth (Agro CS, Česká Skalice, Czech Republic) until reaching the growth stage of DC 13 (three true leaves completed development) when the spot application of metribuzin was done. The substrate was not tested for the presence of herbicide residues. 

A spot application of herbicide metribuzin (C_8_H_14_N_4_OS), (4-amino-6-(l,l-dimethyl)-3-(methylthio)-l,2,4-triazin-5(4H)-one) was performed in leaves, which completed the development and showed no signs of senescence (2nd and 3rd leaf). First, 16 μL of metribuzin (concentrate of 600 g/L, Bayer Garden) was dissolved in 10 mL of distilled water. Then, a 1 μL drop of metribuzin solution (4.48 × 10^−6^ mM of metribuzin) was applied into the centre of leaves (adaxial surface). This concentration corresponds to the recommended concentration used in practice. The plants were after drying of herbicide drop transferred back into the growth chambers for the next four days.

### 2.2. Raman Spectroscopy

Raman imaging was performed on an InVia spectrometer (Renishaw, Wotton-under-Edge, UK) equipped with a Leica confocal microscope. The imaging was undertaken in streamline mode (line focus). The instrument was calibrated to a silicon Raman band at 520.5 cm^−1^. Prior to imaging acquisition, the balance between sufficient Raman signal and heat-induced destruction of the sample was determined. These factors were controlled especially by exposure time, laser power, as well as shape and/or size of the laser spot. The line-by-line mapping used here is advantageous in this regard compared to point-by-point mapping due to its distribution of beam energy over a larger area (see Nasdala et al. [37]). The cut leaf was attached to a 5 mm thick slide using double-sided glue tape to obtain a consistent focal plane. The leaf petiole was put to the bowl-shaped hole (about 5 mm in diameter and 4 mm in depth) in the slide and filled by water with subsequent stabilization by porous plastic foam. Then the leaf’s adaxial surface was immediately subjected to the analysis. For carotenoid imaging, an Ar laser at 514.5 nm wavelength was used with 2.5 mW power at source and 3 s exposure time. A strong signal of carotenoids was obtained due to the resonance Raman effect. Simultaneously, using relatively low laser power and short exposure time allowed the higher number of spectra in the dataset to be obtained within a reasonable time. It is essential here due to possible water-deprivation of the cut leaf. As a result, a relatively large area was scanned at a high spatial resolution. The laser was focused using a 5× magnification Leica objective (NA = 0.12). Datasets of 230,000–250,000 spectra spanning the spectral range 250–2100 cm^−1^ were obtained and processed as described below. Single spectra (five replicates) were extracted from the zones of interest in order to determine temporal variability of absolute intensities and wavenumber positions of ν_1_(C=C) band.

Raman imaging data were processed using Wire 3.4 (Renishaw). First, to reduce the demands upon the PC and enable further processing, the large datasets were truncated in spectral range to the region of interest. In this case it means the region where ν_1_(C=C) modes of carotenoids are located. Then, spectral artifacts due to cosmic rays were removed using the nearest neighbor method, and the spectral noise was filtered through the datasets before map construction. In order to obtain distribution maps of carotenoid signal intensity, the “signal-to-baseline” function was applied for the region 1490–1545 cm^−1^ to construct Raman maps corresponding to the ν_1_(C=C) band expression. The look-up table was set to span 5–95% intensity range (rainbow scale from black to red). For analysis of the shift in wavenumber position of the ν_1_(C=C) band, the spectral datasets were curve-fitted with subsequent application of “peak position”. In that case, the look-up-table was set to span the shift within the wavenumber range 1517.5–1519 cm^−1^ (rainbow scale from black to red). Individual Raman spectra from different map areas were checked, and examples of baseline-corrected spectra extracted from the imaging datasets are presented, maintaining the relative intensities.

### 2.3. Chlorophyll Fluorescence Imaging

ChlF inductions measurements were undertaken using a FluorCam FC 800-O/2020-S open kinetic imaging fluorometer (Photon Systems Instruments spol. s r.o.) through a quenching analysis measuring protocol with continuous actinic red light (617 nm; 600 μmol m^−2^ s^−1^) and saturating pulse (cool white; 2000 μmol m^−2^ s^−1^) after 25 min of dark adaptation. The following parameters related to photosynthetic properties of the treated plants were examined: (i) the maximum quantum yield of PSII photochemistry (*F*v/*F*m = [*F*o − *F*m]/*F*m), (ii) non-photochemical quenching (NPQ = [*F*m − Fm′]/*F*m′). Quenching analysis was measured with a spatial resolution of 1360 × 1024 pixels. The fast fluorescence kinetics protocol was used to determine the probability that a trapped exciton moves an electron into the electron transport chain beyond primary quinone electron acceptors of PSII (1 − *V_J_*). The fast fluorescence protocol required measurement with a reduced spatial resolution (680 pixels × 512 pixels).

### 2.4. Thermal Imaging

An SC 660 thermal imaging camera (FLIR Systems, Wilsonville, OR, USA) was used to measure spatial variability in leaf temperature. The images were taken from top view at the distance 40 cm perpendicular to the leaf. Measurements were taken at the constant air temperature of 25 °C and photosynthetically active radiation 1000 μmol m^−2^ s^−1^ in the growth chamber after 20 min for temperature equilibration. The leaf temperature images were analyzed using ThermaCAM Researcher Professional 2.10 Software (FLIR Systems, Wilsonville, OR, USA). The average temperature of the leaf with applied herbicide was compared to the average temperature of 5 leaves without herbicide application at each time point, to analyze the temperature increase caused by the herbicide effect. 

## 3. Results

### 3.1. Raman Imaging

**Figure 1:**
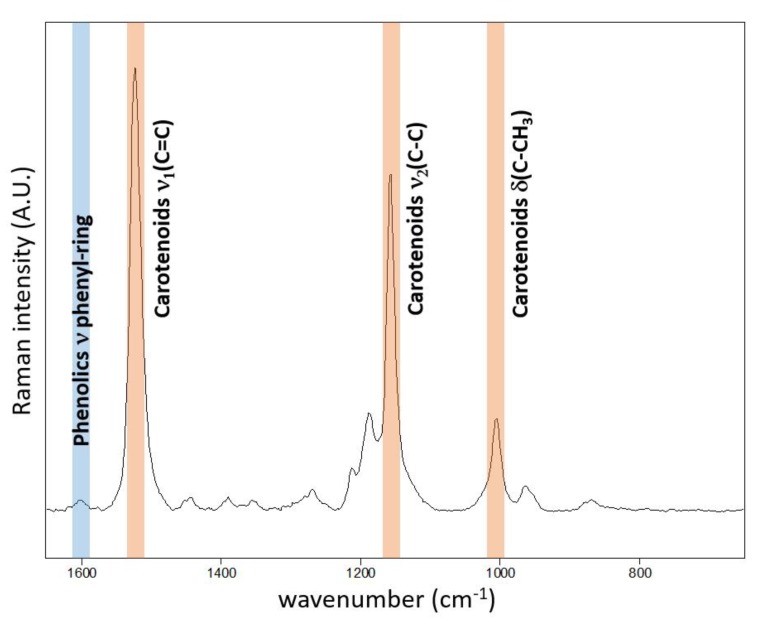
Point Raman spectra obtained at the adaxial surface of *Chenopodium album* leaf using 514.5 nm excitation wavelength. The spectrum is strongly dominated by resonantly enhanced carotenoid features. Nevertheless, weak features attributed to phenolics are also observed in single-point spectra around 1600 cm^−1^.

The typical resonance Raman spectrum obtained on the adaxial surface of the *Chenopodium album* leaf is presented in Figure 1. The corroborative Raman bands of carotenoids within leaves of *Chenopodium album* were detected at 1515–1522, 1154 and 1003 cm^−1^. The integration of the ν_1_(C=C) band occurring at 1515–1522 cm^−1^ was used for the construction of presented Raman maps. 

**Figure 2:**
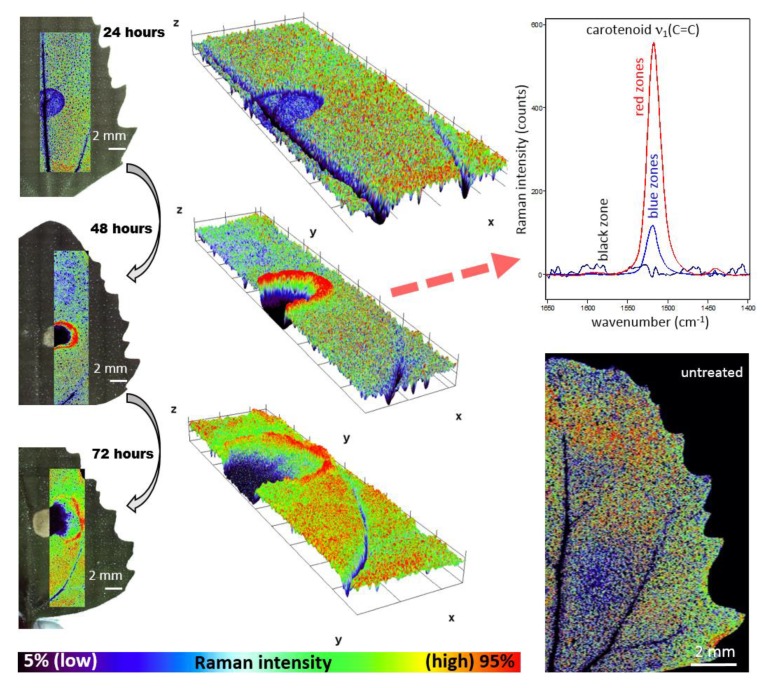
Distribution of Raman signal-to-baseline intensity of the carotenoid ν_1_(C=C) band in leaves of *Chenopodium album*. The area around the herbicide application was scanned 1–3 days after application. The 3D plots represent a visualization of 2D spatial information, with *z*-axis representing the Raman intensity of the ν_1_(C=C) band.

Raman imaging approach revealed local spatial zonality of carotenoid signal distribution. Within 24 h after the application of herbicide to *Chenopodium album*, the decreased intensity of the carotenoid signal was observed in the area of application (Figure 2). It is assigned to the starting destruction of photosystem II, including carotenoids and partial screening of the signal by the dried surface layer of the applied herbicide. After 48 h, the necrosis of leaves was visually observed in the area of application. Raman analysis shows a lack of any Raman features within the necrosis. Importantly, the zonality of increased carotenoid content around the destroyed part of the tissue was observed. Further dispersion of the zone of increased carotenoid signal around the necrotic area was observed 72 h after application (Figure 2).

**Figure 3:**
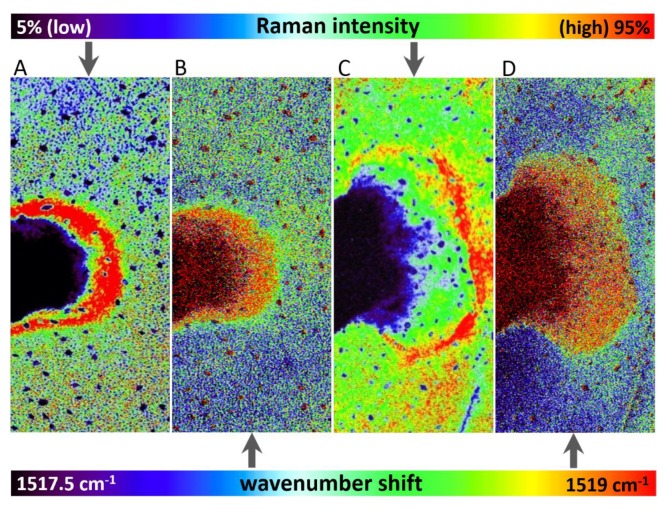
Comparison of Raman signal-to-baseline intensity (**A**,**C**) and band wavenumber position (**B**,**D**) of the carotenoid ν_1_(C=C) band in altered areas of leaves of *Chenopodium album* after 48 h (**A**,**B**) and 72 h (**C**,**D**) of treatment, respectively.

Wavenumber shift was detected after 48 h and 72 h from herbicide application (Figure 3), however, this effect was negligible after 24 h from the application. The depicted images were constructed based on wavenumber shift in the range 1517.5–1519 cm^−1^.

**Figure 4:**
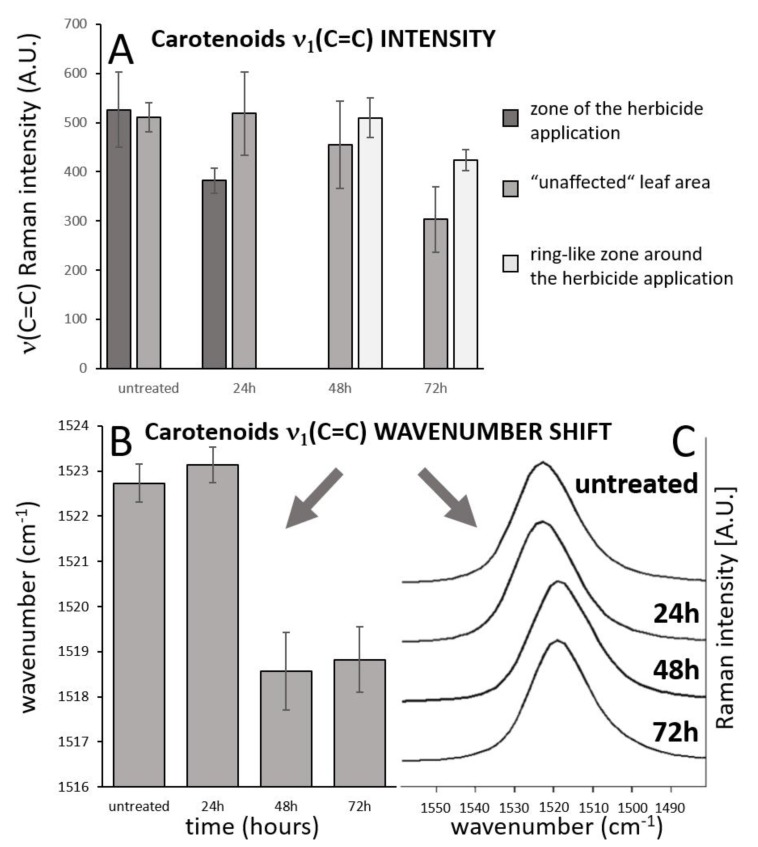
Analysis of temporal changes of the carotenoid ν_1_(C=C) band intensity in the three different zones of the leaf. (**A**). Wavenumber shift of the carotenoid ν_1_(C=C) band with time (**B**,**C**), spectral data come from imaging datasets and represent mean of 5 randomly selected point spectra from the leaf area that is out of the zone of herbicide application and out of the zone of local carotenoid signal enhancement. Error bars represent standard deviations.

Importantly, overall changes in carotenoid Raman spectral features were obtained from the average of five single spectra randomly selected and extracted from the Raman maps. Both changes in Raman intensity as well as in wavenumber position were registered among the treatments. The Raman ν_1_(C=C) band intensity decreased within zones, which are out of the area of herbicide application, which moved slightly away from this spot with the prolonged period after herbicide treatment. In the zones around the necrosis (red-cultured ring-like features in Figure 2 and Figure 3) the decrease was smaller with the treatment duration. A substantial temporally resolved wavenumber shift of the ν_1_(C=C) band was detected from initial values 1522–1523 cm^−1^ detected within zones out of visible herbicide effect in leaves of untreated plants and also leaves 24 h after treatment, towards values 1517–1519 cm^−1^, detected 48 h and 72 h after treatment (Figure 4B,C).

### 3.2. Chlorophyll Fluorescence Imaging

**Figure 5:**
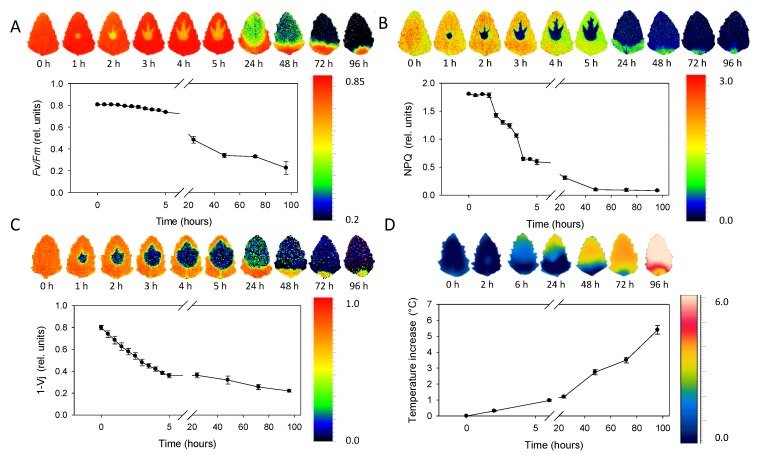
Sequence of chlorophyll fluorescence and thermal imaging of leaves in the span 0–96 h after application. (**A**) The maximum quantum efficiency of PSII (*F*v/*F*m). (**B**) Non-photochemical quenching (NPQ). (**C**) Probability that a trapped exciton moves an electron into the electron transport chain beyond primary quinone electron acceptors of PSII (1 − *V_j_*). (**D**) Temperature increase in comparison to untreated control. Points represent means and error bars standard deviations (*n* = 5).

The sequence of chlorophyll fluorescence images of *Chenopodium album* leaves ranging 0–96 h after the metribuzin application is depicted in Figure 5A–C. The maximum quantum efficiency of PSII (*F*v/*F*m) decreased slowly in the range 0–5 h after application (Figure 5A). Then the decrease became more obvious 24 and 48 h after application. NPQ decreased more rapidly in the range 2–5 h after application with a further decrease after 24 h and approaching minima 48 h after application (Figure 5B). The 1 − *V_j_* parameter decreased rapidly and continuously within the range 0–5 h after herbicide application and just slightly further decreased 24 h and later after application (Figure 5C). Spatial distribution of *F*v/*F*m and NPQ parameters revealed rapid (in hours) movement of herbicide acropetally (in direction to the leaf tip) mainly through main veins. The area affected by herbicide is sharply defined without transition parts. *F*v/*F*m in the area of herbicide effect was reduced approximately by 50% while NPQ by ca 90% already during the first hours after herbicide application. Parameter 1 − *V_j_* showed less evident bonding of the spatial distribution to the main veins, but similarly to *F*v/*F*m and NPQ, it also exhibited acropetal movement within the leaf. 

### 3.3. Thermal Imaging

The average leaf temperature increase compared to leaves without herbicide treatment rose continuously from 0 to values above 5 °C that were detected 96 h after herbicide application (Figure 5D). Similarly to ChlF parameters, the temperature increase was more evident in the upper half of leaf, demonstrating the acropetal movement; however, the infrared thermal imaging was not able to monitor herbicide transport through main veins. The transition between the area affected and non-affected by herbicide application was also less sharply defined with the smooth transition. 

## 4. Discussion

When plants are exposed to high light intensities and the photoprotective dissipation of excess light energy (NPQ) is not efficient, photo-oxidative stress occurs [38]. Production of reactive oxygen species (ROS) is an integrating point of plant response to multiple abiotic and biotic stressors acting either as a signaling or damaging molecule [39]. Photo-oxidative stress can be easily simulated by the application of PSII inhibitors, which can be precisely located and defined in time. It enables us to observe the dynamics and changes in the spatial distribution of biochemical and physiological responses on the leaf or plant level. PSII inhibiting herbicides act by binding to exchangeable quinone site (Q_B_) in the PSII reaction centre, thus blocking electron transport [40]. However, the energy starvation is not a primary cause of the cell and plant death after herbicide exposure. Blocking of electron transport in light-exposed plants leads to the formation of ROS in the reaction center, followed by oxidative stress and protein damage [3]. When the production of ROS is maintained in non-toxic levels, ROS plays important roles as stress signaling molecules inducing several antioxidant and protection mechanisms [41]. ROS signaling is, for example, integrated with accumulation of abscisic acid (ABA) by enhancing ABA biosynthesis or ABA degradation [42] inducing the stomata closure or leaf senescence [43], but ABA also plays a vital role in the induction of defense mechanisms [44].

Plants have evolved several mechanisms of ROS scavenging, including enzymatic and non-enzymatic antioxidants such as ascorbic acid, glutathione, tocopherol, carotenoids, flavonoids or proline [2]. Although the mechanisms are not yet fully understood, it is suggested that ROS signaling is intimately involved in carotenoid biosynthesis by regulating the expression of numerous enzymes such as phytoene desaturase, lycopene β-cyclase or β-carotene hydroxylase [45]. In contrast, high ROS levels induce programmed cell death as an alternative defense program [46]. Programmed cell death is an active process in which cells are selectively eliminated through the involvement of specific proteases and nucleases, and thus no oxidative damage to the neighboring cells is inflicted [46]. A similar cascade of signaling, acclimation and damage responses can be expected for many abiotic and biotic stresses that are associated with increased ROS accumulation [41]. In order to reliably evaluate acclimation processes in plants, the balance between damage and defense, and to understand resistance to abiotic or biotic stress in plants the non-invasive imaging methods allowing dynamic measurement of different processes within the response cascade together with the analysis of the spatial distribution of individual processes are required.

Within this study, the combination of three imaging approaches gave us a spatially resolved insight into the inhibition of PSII, physiological effect on stomatal closure and activation of protective mechanisms of the plant against the PSII inhibition and subsequent oxidative stress. The results of ChlF reflect a time-dependent change of photosynthetic properties and their spatial distribution within the affected leaves. It is evident that different ChlF parameters provide information about dynamics and spatial variability of fast responses to PSII inhibition, which occur in minutes (e.g., 1 − *V_j_*) or minutes to first hours (e.g., NPQ) and also about the slow changes, which occur in hours (*F*v/*F*m). Our data from ChlF imaging also clearly demonstrated the movement of herbicide metribuzin acropetally in direction to the tip of the leaf with preferential flow through the main veins. In addition, these parameters could characterize different photochemical processes ranging from electron transport rate to non-photochemical energy dissipation, which is connected to the conversion of xanthophyll cycle carotenoids [7,47]. However, ChlF itself does not possess any direct spectroscopic evidence of carotenoids [48]. Therefore, combination with Raman spectroscopy, which is a sensitive spectroscopic tool for carotenoid detection, is especially beneficial. Carotenoids are considered to be essential molecules serving in defense against ROS as the main ^1^O_2_ quencher in chloroplasts [49,50,51]. Our results of Raman mapping of carotenoids show three spatially clearly bounded regions of carotenoid distribution. The first region representing the herbicide application area and its very close surroundings showed a fundamental decline in carotenoid content, which is likely caused by programmed cell death. The narrow strip area around the herbicide application spot then revealed a significant increase of carotenoid content compared to both herbicide application area and also the remaining leaf parts. This ring with higher carotenoid content is likely a sign of activation of defense mechanisms. The role of particular carotenoids in the protection of PSII from photoinhibition was studied, e.g., by Schäfer et al. [52] and Kusama et al. [53]. Kusama et al. [53] demonstrated the specific role of echinenone and/or zeaxanthin in the protection of PSII in *Synechocystis*. The authors experimentally proved that the absence of zeaxanthin and echinenone might not accelerate photodamage to PSII but might rather inhibit the repair mechanism of photodamaged PSII. The ability of carotenoids to scavenge radicals was reported to be related to the length of the polyene chain, with higher conjugation being more effective [54]. Increase of conjugation is generally reflected within carotenoid Raman spectra by the shift of the ν_1_(C=C) band towards lower wavenumber positions [17,18], which was demonstrated also in our study 48 h after metribuzin application. Although the shift may reflect various other factors, the carotenoid conjugation has the most significant effect [55]. A similar shift in the ν_1_(C=C) band position was observed within a depth profile of the cyanobacterial cryptoendolithic colony from the extreme sun irradiation conditions of the Atacama Desert, representing wide photoprotection gradient [56]. Here, the slight wavenumber shift towards higher positions within the zone of increased carotenoid intensity was detected, pointing to another mechanism to take place rather than the increase of conjugation. It was reported in the literature, that common xanthophyll C-40 carotenoids with 11 conjugated double bonds exhibit the ν_1_(C=C) band above 1520 cm^−1^, whereas a broad range was reported for β-carotene features that may range also to lower positions (1515–1525 cm^−1^; [17,57,58]). Ruban et al. [59] showed that within xanthophylls, the ν_1_ region was observed at the lowest position for zeaxanthin and shifted towards longer wavenumber with lutein, violaxanthin and neoxanthin. In addition, the selective resonance Raman effect at the used excitation wavelength (514.5 nm) may also play a role in the observed wavenumber shift, enhancing preferentially signal of carotenoids, whose absorption band of an allowed π–π* transition coincides the most intensively with a 514.5 nm line. From the xanthophyll cycle carotenoids, zeaxanthin Raman features are preferentially resonantly enhanced when excited by a 514.5 nm laser wavelength [60].

Given that the stomatal response to stress is largely coordinated by the level of ABA [42], one of the manifestations of stress in leaves is stomata closure resulting in reduced photosynthetic CO_2_ assimilation and also decline in transpiration. Reduced transpiration limits the plant cooling and stressed plants exhibit a higher temperature than stress-free plants [61]. The development of infrared thermal imaging techniques made possible to monitor spatial and dynamic changes in stomatal responses to stress stimuli [62], however, infrared thermal imaging has not been yet used to monitor dynamics and spatial distribution of herbicide effect at the leaf and plant level. Our results show, similarly to chlorophyll fluorescence imaging, that the herbicide effect on stomata closure spreads acropetally from the site of herbicide application, which results in a temperature increase particularly in the area from the place of herbicide application to the leaf tips. The effect of herbicide on stomata closure measured as temperature increase compared to control was gradual during 96 h of the experiment. The regulation of stomatal response to ABA is mediated by a complex symphony of intracellular signaling in which nitric oxide (NO) plays a central role [63]. ABA triggers NO generation, which in turn together with H_2_O_2_ induce both antioxidant defenses and stomatal closure [64]. The resulting stomatal response is, therefore determined by the balance between induced damage and defense reactions. Our results show that the plant initiates the protective mechanisms outside the place of herbicide application and thereby slow down the stomata closure.

## 5. Conclusions

- The combination of Raman, chlorophyll *a* fluorescence and thermal imaging allowed us to monitor the cascade of damage and defense responses following the application of PSII inhibiting herbicide with high spatial resolution, and also proved the usefulness for the monitoring of numerous abiotic and biotic stresses showing a similar cascade of response.
- ChlF imaging allowed us to monitor time-dependent metribuzin transport in acropetal direction through main veins and demonstrated the ability to distinguish between fast processes at the level of electron transport (1 − *V_j_*) or non-photochemical energy dissipation (NPQ) and slower effect on maximum efficiency of PSII photochemistry (*F*v/*F*m).
- The high-resolution resonance Raman images of leaves show zones of local increase of carotenoid content relative to the rest of the leaf 48 and 72 h after the herbicide application, surrounding the damaged tissue in the zone of herbicide application, which is an indication of activation defense mechanisms in leaf.
- The substantial time-dependent variability in the carotenoid band position (from 1523 to 1517 cm^−1^) and also spatial variability of the band position after 48 h and 78 h (from 1517.5 to 1519 cm^−1^) indicates structural changes in the carotenoid composition.
- The increase of leaf temperature in the region surrounding the spot of herbicide application and expanding acropetally in the direction to the leaf tip proved the metribuzin effect on stomata closure, which was relatively slower compared to other responses.
